# Hematopoietic cell transplantation for myeloid/NK cell precursor acute leukemia in second remission

**DOI:** 10.1002/ccr3.1506

**Published:** 2018-04-10

**Authors:** Yusuke Noguchi, Daisuke Tomizawa, Haruka Hiroki, Satoshi Miyamoto, Mari Tezuka, Reiji Miyawaki, Mari Tanaka‐Kubota, Tubasa Okano, Chika Kobayashi, Noriko Mitsuiki, Yuki Aoki, Kohsuke Imai, Michiko Kajiwara, Hirokazu Kanegane, Tomohiro Morio, Masatoshi Takagi

**Affiliations:** ^1^ Department of Pediatrics and Developmental Biology Graduate School of Medicine Tokyo Medical and Dental University Yushima 1‐5‐45, Bunkyo‐ku Tokyo Japan; ^2^ Division of Leukemia and Lymphoma Children's Cancer Center National Center for Child Health and Development Okura 2‐10‐1, Setagaya‐ku Tokyo Japan; ^3^ Department of Pediatrics Ehime Prefectural Central Hospital Kasuga 83 Matsuyama Ehime Japan; ^4^ Department of Pediatrics Matsuyama Red Cross Hospital Bunkyo‐cho Matsuyama Ehime Japan; ^5^ Department of Pediatrics Oncology National Cancer Research Center Tsukiji 5‐1‐1, Chuo‐ku Tokyo Japan; ^6^ Department of Transfusion Medicine Graduate School of Medicine Tokyo Medical and Dental University Yushima 1‐5‐45, Bunkyo‐ku Tokyo Japan

**Keywords:** Hematopoietic cell transplantation, L‐Asparaginase, myeloid/natural killer cell precursor acute leukemia

## Abstract

Myeloid/natural killer cell precursor acute leukemia (MNKPL) is a rare leukemia subtype characterized by a high incidence of extramedullary infiltration. No appropriate treatment strategy has so far been developed. Acute myelogenous leukemia‐type chemotherapy combined with L‐Asparaginase is an effective treatment for MNKPL. Hematopoietic cell transplantation is a second option in refractory cases.

## Introduction

Myeloid/natural killer (NK) cell precursor acute leukemia (MNKPL) is a rare type of leukemia prevalent in Asia. MNKPL is a distinct entity in that it differs from myeloid/NK cell leukemia (MNKL) and blastic NK cell lymphoma/leukemia in terms of both morphology and immune type. MNKPL is characterized by marked extramedullary involvement, immature lymphoblastoid morphology without myeloperoxidase (MPO) reactivity, a CD7^+^/CD33^+^/CD34^+^/CD16^−^/CD15^−/+^/HLA^−^DR^+^ phenotype, myeloid chemosensitivity, and a poor prognosis. By contrast, MNKL shows no extramedullary involvement, a HLA‐DR^−^/CD33^+^/CD16^−^/CD34^−/+^ phenotype, myeloid chemosensitivity, and a good prognosis. Because MNKPL is so rare, no appropriate therapeutic strategy has been established, making it hard to undertake systemic clinical trials. Therefore, accumulation of clinical observations and retrospective cohort studies will provide important information that can be used to develop future therapies.

## Case Presentation

**Figure 1:**
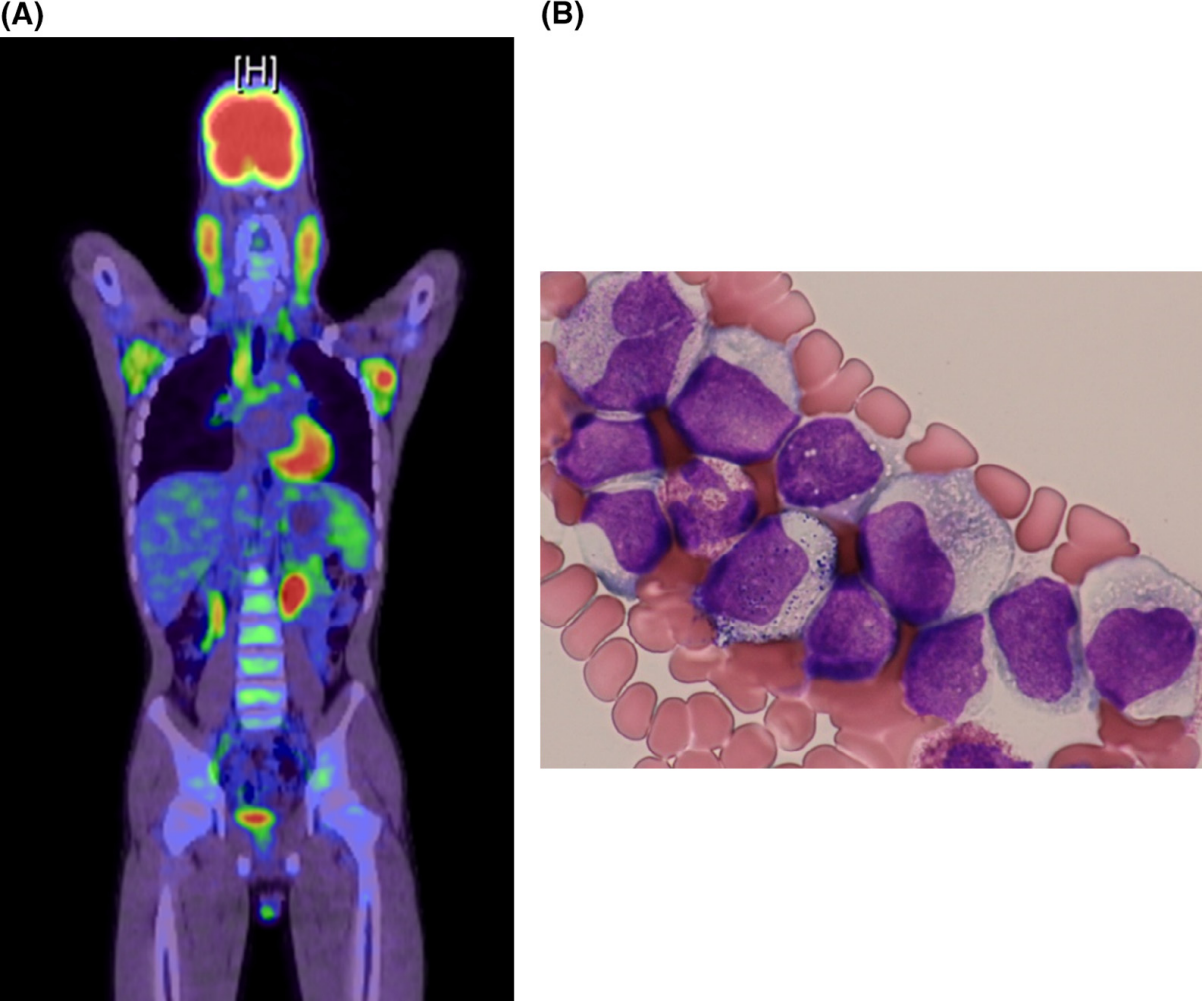
(A) ^18^F‐FDG PET/CT shows hypermetabolic lesions in multiple lymphoid organs, especially the bilateral cervical and axillar lymph nodes. FDG, fluorodeoxyglucose; PET/CT, positron emission tomography/computed tomography. (B) Wright–Giemsa staining of a bone marrow aspiration smear. Blast cells are relatively large and harbor fine azurophilic granules in the cytoplasm.

**Figure 2:**
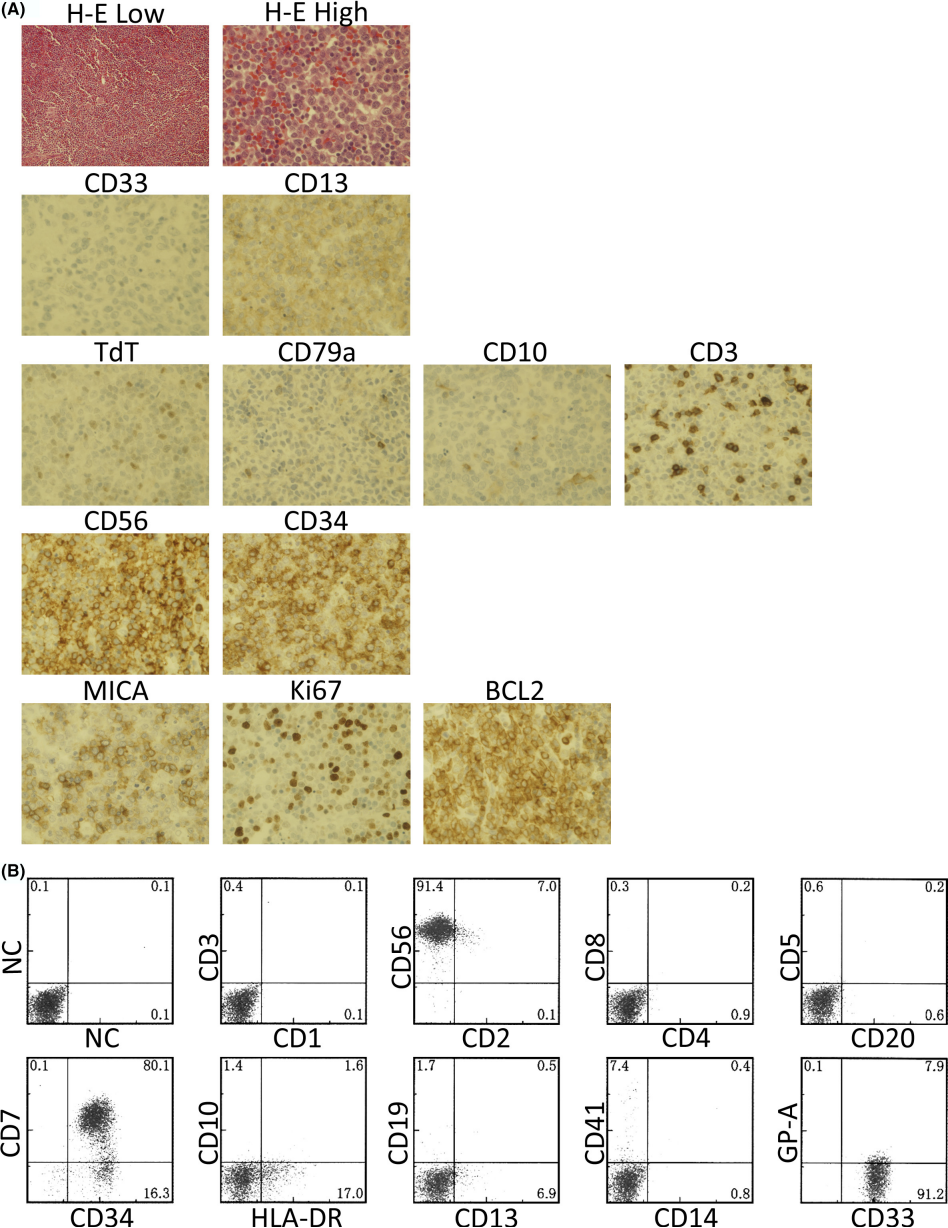
(A) Photomicrograph of a lymph node specimen. H‐E = hematoxylin–eosin staining. Low = lower magnification, High = higher magnification. (B) Dot blot graph of flow cytometric data. The CD45 dull population was analyzed using the indicated antibody. NC = normal control IgG.

A 13‐year‐old Japanese boy presented to our hospital with fever, fatigue, and bilateral cervical lymphadenopathy. His family history did not reveal any health problems pertinent to his illness. A PET scan confirmed massive lymphadenopathy (Fig. 1A). Bone marrow aspiration revealed that 40% of the bone marrow cells comprised MPO‐negative blast cells (Fig. 1B), which were CD56^+^/CD7^+^/CD33^+^/CD34^+^/HLA‐DR^+^ (Table 1, Fig. 2A and B). A lymph node biopsy also revealed massive infiltration by blast cells^+^ (Fig. 2A). Immunohistochemical staining showed strong positivity for CD56, CD34, and BCL2, and moderate positivity for CD33 and MICA. Laboratory findings are shown in Table 1.

**Table 1:** Laboratory data on initial diagnosis

**Peripheral blood analysis**				**Blood chemical analysis**			
WBC		1.7 × 10^9^/L (Blasts, 0%)		TP		8.4 g/dL	
RBC		4.49 × 10^12^/L		BUN		4 mg/dL	
Hb		13.7 g/dL		Cre		0.19 mg/dL	
HCT		38.6%		LDH		408 U/L	
Platelet		1.88 × 10^11^/L		AST		51 U/L	
Reticulocyte		14.8‰		ALT		30 U/L	
				CRP		1.62 mg/dL	
				sIL2R		992 U/mL	
**Bone marrow examination**				**Cytogenetic analysis**			
Nuclear cell count		2.6 × 10^10^/L		47,XY, +10 [7/20]			
Megakaryocytes		15/*μ*L					
Blasts		36.3%					
Blast cells were MPO‐negative/esterase‐negative.							
**Flow cytometry analysis**							
**B cells**		**T/NK cells**		**Myeloid cells**		**Other**	
CD19	2.2%	CD2	7.1%	cMPO	21.9%	CD34	98.2%
CD20	0.8%	CD5	0.8%	CD13	7.4%	CD38	91.5%
CD10	3.0%	CD3	0.6%	CD33	99.1%	HLA‐DR	4.9%
CD7	80.2%	CD117	22.0%				
CD56	98.5%	CD11b	97.1%				

MPO, myeloperoxidase.

**Figure 3:**
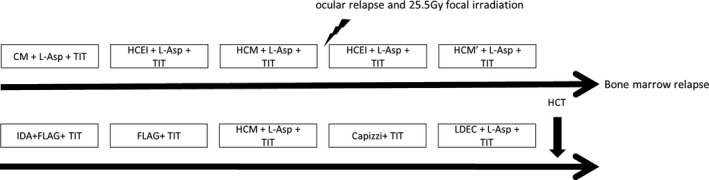
Clinical course of the patient. CM: cytarabine, 200 mg/m^2^ × 7 days + mitoxantrone, 5 mg/m^2^ × 5 days; HCEI: cytarabine, 3 g/m^2^ q12 h × 3 days + etoposide, 100 mg/m^2^ × 5 days + idarubicin, 10 mg/m^2^; HCM: cytarabine, 2 g/m^2^ q12 h × 3 days + mitoxantrone, 5 mg/m^2^ × 5 days; L‐asparaginase: 10,000 U/m^2^; TIT: methotrexate, 12 mg + cytarabine, 30 mg + hydrocortisone, 25 mg; IDA‐FLAG: IDA, 10 mg/m^2^ × 3 days + fludarabine, 30 mg/m^2^ × 5 days + cytarabine, 2 g/m^2^ q12 h × 3 days + G‐CSF, 5 *μ*g/kg × 6 days; Capizzi: cytarabine 3 g/m^2^ q12 h × 3 days + L‐Asp, 10,000 U/m^2^ × 5 days; LDEC: AraC, 20 mg/m^2^ × 1 day + etoposide, 30 mg/m^2^ × 1 day.

The patient was diagnosed with MNKPL, for which there is no clinically evaluated chemotherapeutic strategy. A previous report suggests that acute myelogenous leukemia (AML)‐type chemotherapy may be of benefit 1, whereas other studies suggest that L‐asparaginase (L‐Asp) is effective against NK/T‐cell‐type lymphomas 2, 3. Therefore, we used AML‐type chemotherapy combined with L‐Asp (10,000 U/m^2^ × 5 times per cycle; five cycles in total). Induction chemotherapy led to complete remission (CR). However, the leukemia relapsed (ocular involvement) after two rounds of intensification chemotherapy 4. After local ocular irradiation and four rounds of intensification treatment, second CR was achieved and the patient was discharged. However, 1 year after cessation of chemotherapy, pancytopenia and cervical lymph node swelling re‐appeared. Bone marrow aspiration revealed bone marrow relapse. Therefore, the patient received idarubicin (IDA) combined with FLAG induction therapy, followed by one cycle of FLAG, Capizzi, and LDED intensification therapy. Second CR was again achieved. The patient then underwent hematopoietic cell transplantation (HCT) with 8/8 HLA‐matched unrelated bone marrow. The conditioning regimen comprised total body irradiation (TBI; 12 Gy) and melphalan (60 mg/m^2^/day for 3 days). Tacrolimus (0.02 mg/kg/day) and methotrexate (MTX; 15 mg/m^2^ on Day 1 and 10 mg/m^2^ on Days 3, 6, and 11) were used for graft versus host disease (GVHD) prophylaxis. Engraftment was achieved by Day 21. Grade I GVHD was observed. Regimen‐related toxicity was moderate. The patient stayed in remission for 2 years after HCT (Fig. 3).

## Discussion

Several case reports describing MNKPL have been published. It should be refrain that MNKPL is different entity to be distinguished from MNKL. MNKPL has a poor prognosis, whereas the prognosis for MNKL is much better. MNKPL is characterized by CD34 antigen‐positivity and extramedullary involvement 1, 5. The 2016 revision to the World Health Organization classification of myeloid neoplasms and acute leukemia does not mention MNKPL, but it may be categorized as AML with minimal differentiation or mixed phenotype acute leukemia, and not otherwise specified rare types (MPAL NOS rare types) 6.

MNKPL shows a better therapeutic response to AML chemotherapy regimens than to ALL regimens 1, and L‐Asp as a single agent is an effective treatment for relapsed/refractory NK/T‐cell lymphomas 2, 3. Based on these observations, L‐Asp has been incorporated into AML‐type regimens used to treat several cases, including our own.

A literature review identified 13 pediatric (less than 20 years of age) MNKPL cases (Table 2). The age of onset was between 0.9 and 19 years of age (mean, 8.3 years), and nine of 13 cases were male (69%). Six cases survived and were disease free (observation period, 24–63 months), and seven cases received AML‐type chemotherapy during the induction, consolidation, or intensification phase. On the other hand, Guan et al. reported that the ALL‐type regimen was effective in three MNKPL cases when using cytarabine, mitoxantrone, etoposide, (an agent used for the treatment of AML) and L‐Asp during the consolidation and intensification phases 7. Among 13 pediatric cases, three cases received AML‐type chemotherapy combined with L‐Asp, eight cases received allogenic HCT, and one case received autologous HCT. Among those who received allogenic HCT, two cases stayed in remission (observation period between 24 and 46 months). The three patients that received AML‐type chemotherapy combined with L‐Asp (one of whom also received HCT) survived. In our case, initial chemotherapy comprised AML‐type chemotherapy combined with L‐Asp, followed by FLAG‐IDA and a FLAG regimen (re‐induction therapy) after relapse. A previous study reported that the FLAG regimen is an effective second‐line therapy for MNKPL 8. Our experience and previous observations suggest that AML‐type chemotherapy combined with L‐Asp is an effective treatment for MNKPL. A FLAG‐based regimen might also be effective in refractory cases. We identified 14 adult cases (Table 2). Age of onset was between 21 and 63 years of age. Nine cases received myeloid‐based chemotherapy, and three cases received HCT. Among those who received HCT, one case survived without disease free for more than 2 years. Among the seven cases without HCT, none survived. Our literature review did not clarify whether or not HCT benefits children in first remission. These observations suggest that, at least in adults, HCT must be performed in first remission.

**Table 2:** Literature review of MNKPL cases

Case	Age	Sex	Treatment	Type of HCT	Outcome	Reference
Child cases						
1	17	M	AML, ALL, VP16 + AraC	Haplo BMT	Relapse after 20 months	9
2	12.5	M	ALL+MIT, AraC, VP16		63 months	7
3	8.5	M	ALL+MIT, AraC, VP16		38 months	7
4	1.3	M	ALL+MIT, AraC, VP16		26 months	7
5	3.8	M	ALL+MIT, AraC, VP16		7 months^a^	7
6	5	F	AML+L‐Asp		40 months	10
7	6	F	AML+L‐Asp	CBT	46 months	11
8	1	M	AML, ALL	HLA mis related BMT	7 months after HCT^a^	12
9	14	F	AraC, ADR, VP16	Sib BMT	ND	13
10	18	M	ALL	Sib BMT	Relapse after 1 month^a^	14
11	2	M	ALL, AML+L‐Asp	CBT	24 months	3
12	0.9	F	VCR, ADR, CPM	Auto BMT	3 months^a^	15
13	19	M	CHOP+L‐Asp, DCVP	Related BMT	19 months^a^	5
Adult cases						
1	63	M			Before chemotherapy^a^	16
2	21	M	DOAP, IDA, AraC, FLAG		ND	8
3	74	F			ND	17
4	62	M			ND	17
5	34	M	MIT, AraC, VP16 (after HCT)	Unrelated BMT	24 months	18
6	37	F	AraC, IDA		1 month^a^	19
7	36	M	MIT, AraC, VP16	Unrelated PBSCT	Donor derived MDS at 7 months^a^	20
8	34	M	AraC, IDA		ND	21
9	34	M	CHOP, DCVP		17 months^a^	5
10	46	M	DCVP		4 months^a^	5
11	54	M	DCMP		30 months^a^	5
12	29	F	DCMP	Sib BMT	19 months due to GVHD^a^	5
13	48	M	ALL		41 months^a^	5
14	59	M	Low dose AraC, DCVP		11 months^a^	5

HCT, hematopoietic cell transplantation; AML, AML‐type chemotherapy; ALL, ALL‐type chemotherapy; AraC, cytarabine VP16, etoposide; MIT, mitoxantrone; L‐Asp, L‐asparaginase; ADR, adriamycin; CPM, cyclophosphamide; CHOP, CPM + ADR + vincristine (VCR) + prednisolone (PSL); DOAP, daunorubicin (DNR) + VCR + AraC + PSL; DCMP, daunorubicin (DNR) + AraC + 6‐mercaptopurine + PSL; DCVP, DNR + AraC + VCR + PSL; BMT, bone marrow transplantation; Haplo BMT, haploidentical matched BMT; CBT, cord blood transplantation; PBSCT, peripheral blood stem cell transplantation; HLA, human leukocyte antigen; sib, sibling; mis, mismatch; auto, autologous.

a Dead.

The incidence of MNKPL is low. Therefore, no systemic clinical trial to develop an appropriate therapeutic approach has been undertaken. In this case, collection of case reports is essential. In addition, international collaborations will speed up progress toward developing a standard treatment plan.

## Authorship

YN: drafted the manuscript. YN, DT, HH, SM, MariT, RM, MTK, TO, CK, NM, and YA: participated in patient care and data correction, and helped with the literature review. MK: managed HCT. KI, MK, HK, TM, and MT: supervised manuscript preparation and performed proofreading of the final manuscript.

## Conflict of Interest

None declared.
